# Study protocol for a pragmatic randomised controlled trial in Belgian primary care and hospital settings on the effectiveness of an eHealth self-management support programme consisting of pain education and coaching of activity needs in breast cancer survivors with persistent pain: the PECAN trial

**DOI:** 10.1136/bmjopen-2025-099241

**Published:** 2025-08-22

**Authors:** An De Groef, Lore Dams, G Lorimer Moseley, Lauren C Heathcote, Louise K Wiles, Mark Catley, Anna Vogelzang, Peter Hibbert, Bart Morlion, Marthe Van Overbeke, Emma Tack, Sophie Van Dijck, Nele Devoogdt, Ceren Gursen, Annick L De Paepe, Michel Mertens, Josefien van Olmen, Lander Willem, Wiebren Tjalma, Ines Nevelsteen, Patrick Neven, Rani Vanhoudt, Davina Wildemeersch, Femke De Backere, Steffen Fieuws, Geert Crombez, Mira Meeus

**Affiliations:** 1 Department of Rehabilitation Sciences, KU Leuven – University of Leuven, Leuven, Belgium; 2 Department of Rehabilitation Sciences, University of Antwerp, Antwerp, Belgium; 3 Department of Physical and Rehabilitation Medicine, University Hospitals Leuven, Leuven, Belgium; 4 Innovation, Implementation & Clinical Translation (IIMPACT) in Health, University of South Australia, Kaurna Country, Adelaide, South Australia, Australia; 5 Health Psychology Section, Department of Psychology, Institute of Psychiatry, Psychology, and Neuroscience, King’s College London, Londen, UK; 6 Australian Institute of Health Innovation, Macquarie University, Sydney, New South Wales, Australia; 7 Department of Cardiovascular Sciences, Section Anaesthesiology & Algology, KU Leuven – University of Leuven, Leuven, Belgium; 8 Department of Experimental-Health Psychology, Faculty of Health Psychology, Ghent University, Ghent, Belgium; 9 Research School CAPHRI, Department of Rehabilitation Medicine, Maastricht University, Maastricht, The Netherlands; 10 Department of Family Medicine and Population Health (FAMPOP), University of Antwerp, Antwerp, Belgium; 11 Multidisciplinary Breast Clinic, Antwerp University Hospital, Antwerp, Belgium; 12 Faculty of Medicine and Health Sciences, University of Antwerp, Antwerp, Belgium; 13 Department of Surgical Oncology, University Hospital Leuven, Leuven, Belgium; 14 Department of Gynaecological Oncology, University Hospital Leuven, Leuven, Belgium; 15 Multidisciplinary Pain Centre (PCT), University Hospital Antwerp, Wilrijk, Belgium; 16 IDLab, Department of Information Technology - imec, Ghent University, Ghent, Belgium; 17 Department of Public Health, Interuniversity Center for Biostatistics and Statistical Bioinformatics, KU Leuven, Leuven, Belgium

**Keywords:** eHealth, Self-Management, ONCOLOGY, REHABILITATION MEDICINE, PAIN MANAGEMENT

## Abstract

**Introduction:**

Persistent pain after finishing breast cancer treatment is a common and disabling problem. The current state-of-the-art pain management advocates, in addition to biomedical (non-)pharmacological approaches, a biopsychosocial rehabilitation approach to address persistent pain, combining pain science education with promoting an active lifestyle through self-regulation techniques. We propose testing an innovative eHealth self-management support programme for this purpose in the breast cancer population with persistent pain after finishing cancer treatment. This delivery mode is believed to reduce barriers to pain self-management by providing timely, safe and cost-effective assistance addressing the biopsychosocial needs of patients. Utilising a chatbot format, the eHealth programme delivers pain science education and promotes physical activity (PA), personalised through decision-tree-based algorithms to support pain self-management. The programme aims to empower patients with understanding, coping skills and self-management techniques to reduce pain-related disability and enhance participation in daily life. The primary objective is to determine programme effectiveness compared with (1) usual care (superiority) and (2) a similar face-to-face pain self-management support programme (non-inferiority).

**Methods and analysis:**

A pragmatic, three-arm randomised controlled trial was started in April 2024 at the University Hospitals of Antwerp and Leuven and primary care settings in Belgium. Participants are breast cancer survivors with persistent pain after finishing cancer treatment. Two hundred seventy participants will be randomised to one of three trial arms: (1) eHealth self-management support programme, (2) usual care or (3) a face-to-face self-management support programme. The ‘eHealth self-management support programme’ begins with a pain science education (PSE) module to initially convey key pain-related concepts and provide personalised pain management tips. Then, the programme progresses to daily activity planning to promote an active lifestyle. Guided by the Health Action Process Approach (HAPA) model, participants set and review daily activity goals and track progress. The eHealth self-management programme uses a chatbot and is accessible on any digital device. The ‘usual care programme’ involves sending the participants a study-specific brochure by postal mail and does not include any formal PSE and/or PA programmes. They may pursue or continue self-initiated care. In Belgium, usual care primarily involves pharmacological treatment, general advice on PA and the provision of informational brochures. The ‘face-to-face self-management support programme’ mirrors the eHealth intervention, combining PSE with PA coaching. It starts with three individual sessions with a trained physical therapist for biopsychosocial assessment and PSE, followed by six sessions on goal setting and active lifestyle coaching. The educational content is delivered both verbally and in written form. The primary outcome will be pain-related disability 6 months after baseline assessment. As a key secondary outcome, the effect on pain beliefs and attitudes will be investigated after the educational part of the eHealth and face-to-face programme (ie, at 6 weeks after baseline). Other secondary outcomes related to other dimensions of pain and physical-, psychosocial- and health-economic outcomes will be assessed at 12 weeks and 6 and 12 months after baseline as well.

**Ethics and dissemination:**

The study will be conducted in accordance with the Declaration of Helsinki (2024). The protocol has been approved by the ethical committee of the University Hospitals of Leuven and Antwerp. Results will be disseminated via peer-reviewed scientific journals and presentations at congresses. Ethical Committee of the University Hospitals Leuven and Antwerp: BUN B3002023000132.

**Trial registration number:**

ClinicalTrials.gov Identifier: NCT06308029.

**STRENGTHS AND LIMITATIONS OF THIS STUDY**

- This pragmatic trial includes broad eligibility (all ages, treatments, sexes/genders) and recruits from both primary care and hospitals, supporting strong external validity.
- Designed by an international, interdisciplinary team, the project is grounded in strong evidence and theory.
- A process evaluation is included to inform future scale-up decisions.
- External validity is limited by being conducted in a single country and requiring Dutch proficiency, potentially excluding some groups.
- The trial is powered (≥80%) for superiority and non-inferiority comparisons, but not necessarily for the combined endpoint.

## Introduction

 Breast cancer is the most prevalent cancer, accounting for over 33% of all cancers diagnosed globally.^1^ Advances in cancer screening and treatment have significantly increased survival rates, resulting in a growing population of breast cancer survivors. However, many survivors experience long-term, debilitating effects from the disease and its treatments, particularly persistent pain.^2^,^4^ A recent meta-analysis found that 45% of breast cancer survivors report persistent pain after completing primary treatments.^4^

Current cancer pain management predominantly relies on pharmacological approaches, including anti-neuropathic drugs which often have limited efficacy and some with significant side effects.^5^ Complete pain relief is rare with pharmacotherapy; most patients achieve only partial improvement, typically a 30%–50% reduction in pain intensity. In this, particularly opioids can be associated with significant side effects.^5^ Therefore, a more comprehensive approach to pain management is needed.

Chronic pain management in non-cancer populations has moved beyond medication-based approaches.^6^,^10^ Education, active and psychological therapies, and self-management skills are frontline interventions for persistent pain, as endorsed by the clinical guidelines.^6^,^10^ Modern Pain Science Education (PSE) aims to improve understanding of ‘how pain works’, including the biological basis of pain’s multifactorial character, the notion of variable sensitivity and the ‘over-protective’ nature of persistent pain.^11^,^13^ Also, contemporary PSE is grounded in educational and adult learning science principles, moving away from didactic teacher-centred methods or ‘information provision’ approaches.^14^ This approach empowers individuals by improving their understanding of pain, which can lead to better self-management and decision-making regarding their treatment. Meta-analyses have shown that PSE reduces pain, disability, pain catastrophising and kinesiophobia in adults with chronic musculoskeletal pain.^15 16^ For pain after cancer treatment, educational interventions show modest short-term effects on quality of life, particularly in global and emotional aspects, but not for pain in particular.^17^,^20^

Physical Activity (PA) is another crucial component in managing chronic non-cancer pain.^6^,^1021^ It can reduce pain sensitivity by its beneficial effects on the central nervous system, autonomic nervous system, immune system and musculoskeletal system.^22^,^25^ However, misconceptions about pain and PA often prevent individuals from adopting active lifestyles. PSE can prime patients for PA programmes by addressing barriers to behaviour change and bridging the intention-behaviour gap, enabling them to adopt an active lifestyle. This approach is effective in non-cancer populations.^1526^,^31^ Adopting an active lifestyle may require a behavioural change in which self-regulation techniques have proved useful.^32^ To our knowledge, no research has yet combined PSE with a PA behavioural intervention based on self-regulation techniques to support pain self-management in the cancer population.^33^

Implementing combined PSE and PA interventions presents challenges for patients and for the healthcare system. These challenges include the resource-intensive nature of face-to-face rehabilitation programmes, the burden of long-term medical follow-up, stress and inconvenience related to healthcare provider visits, and the need for personalised approaches tailored to individual patients' needs.^15 34 35^ Additionally, accessibility issues, such as mobility limitations and time constraints, limit the coverage of traditional face-to-face programmes. Integration of digital health interventions,^36^ including for pain management,^37^ is now being actively encouraged as a sustainable approach. eHealth interventions can deliver tailored information to large populations cost-effectively, offering widespread accessibility. eHealth self-management support programmes can support individuals to manage their symptoms, treatment and lifestyle changes. In their design, these programmes offer the opportunity to personalise information, advice and skills according to decision-making algorithms. Studies testing chatbot-based eHealth interventions in breast cancer populations and chronic pain settings report high patient engagement, satisfaction and usability, suggesting that chatbots can serve as effective, scalable tools for delivering personalised support outside traditional care settings.^38^,^40^ Evidence suggests that such programmes can positively impact both physical and psychological outcomes in chronic non-cancer pain.^3541^,^44^ However, little evidence is currently available for this approach to improve pain-related disability in the cancer population. A systematic review on effects of eHealth self-management interventions in the breast cancer population found only two uncontrolled studies that demonstrated significant decreases in pain intensity over time. Also, these studies did not involve eHealth interventions that offered tailorable content; their observed effects were not sustained over the long term.^35^

Given all this, the proposed project aims to evaluate the effectiveness of an automated and personalised eHealth self-management support programme for reducing pain-related disability in breast cancer survivors. This programme uses an innovative chatbot format to combine PSE with self-regulation techniques to promote PA after breast cancer, that is, the PECAN trial which stands for ‘Pain Education and Coaching of Activity Needs in breast cancer survivors’.

### Objectives

The primary objective of the study is to determine the effectiveness of an eHealth self-management support programme for persistent pain after breast cancer treatment on pain-related disability at 6 months after baseline, as compared with:

1. Usual care (ie, superiority of the eHealth self-management support programme).
2. A self-management support programme delivered face-to-face in a physical therapy setting (ie, non-inferiority of the eHealth self-management support programme).

The secondary objectives of this study are to:

1. Determine the change in pain beliefs and attitudes at 6 weeks (ie, after the PSE component but before the PA component of the eHealth and face-to-face programme) (key secondary outcome).
2. Examine if the eHealth self-management support programme has a relative benefit for other secondary biopsychosocial outcome parameters that have been aligned with the programme theory, encompassing pain-related, physical and psychosocial outcomes assessed at 12 weeks, 6 months and 12 months.
3. Estimate its cost-effectiveness at 6 and 12 months.

Parallel to the effectiveness trial, a process evaluation will be performed to assess the implementation, the contextual determinants of implementation and the potential for scale-up.

## Methods and analysis

Described according to the Standard Protocol Items: Recommendations for Interventional Trials (SPIRIT) guidelines (http://www.spirit-statement.org/protocol-version/).

### Trial design and study setting

The trial is a parallel, three-arm pragmatic randomised study, approved by the Ethics Committees of the University Hospitals Leuven and Antwerp (BUN B3002023000132) on 30 January 2024. The first participant was enrolled on 26 May 2024. Recruitment is expected to continue until January 2028, resulting in the primary completion in June 2028. Given a 1-year follow-up, the anticipated study completion date is January 2029. A schedule of the trial is presented in table 1.

**Table 1:** Schedule of enrolment, interventions and assessments of the PECAN trial

Timepoint	Study period							
	Enrolment		Allocation	Post-allocation				
	-t_2_ Referral	-t_1_ telephone consult	0	t_1_ baseline	t_2_ 6 weeks after baseline	t_3_ 12 weeks after baseline	t_4_ 6 Mo after baseline	t_5_ 12 Mo after baseline
Enrolment								
Eligibility screen	X							
Informed consent		X						
Randomisation			X					
Allocation			X					
Interventions								
				Education	PA coaching			
eHealth intervention			n=90	x	x			
Face-to-face intervention			n=90	x	x			
Usual care			n=90					
Assessments								
Pain-related disability (primary outcome)*				X		X	X†	X
Pain-related outcomes, including pain beliefs and attitudes (key secondary outcome)*				X	(X)†	X	X	X
Physical outcomes*				X		X	X	X
Psychosocial outcomes*				X		X	X	X
Health economic outcomes*							X	X

* See online supplemental appendix C for details on the content of the different assessments at each point in time.

† Primary endpoint of the trial.

† The key secondary outcome ‘pain beliefs and attitudes’ is the only outcome collected at 6 weeks.

PA, physical activity; PECAN, Pain Education and Coaching of Activity Needs in breast cancer survivors.

### Patient and public involvement in trial design

An advisory board with different interest holders, including Belgian and Flemish patient organisations (Kom Op Tegen Kanker, Stichting Tegen Kanker, ThinkPink and Envie), the Professional Association for physical therapists and general practitioners, and the National Institute for Health and Disability Insurance, was consulted during the initial grant preparation and trial setup. The feasibility, acceptability and utility of the eHealth approach was tested with consumers with lived or living experience of persistent pain after treatment for breast cancer, in collaboration with international partners within the authorship team.^45^

### Eligibility criteria

People with primary breast cancer who have undergone axillary surgery (axillary lymph node dissection or sentinel node biopsy, unilateral or bilateral) are eligible. Participants must be non-metastatic and have finished their primary treatment with curative intent at least 3 months before participating in the study. However, adjuvant hormonal therapy and immunotherapy are allowed. Additionally, participants must report to their healthcare provider persistent pain over the past 3 months that interferes with their daily activities. Given the pragmatic nature of this trial, self-report of persistent pain will be used as the definition for this eligibility criteria. Participants will be excluded from the study if they are unable to participate for the entire duration of the study, are mentally or physically unable to participate, do not have access to a digital device, do not speak/understand Dutch or have previously participated in a PSE programme.

### Participant screening, recruitment and consent

Participants experiencing persistent pain after breast cancer treatment are recruited in the University Hospitals of Leuven and Antwerp, as well as from general practitioners’ practices in Flanders and through self-referral. Following referral, an initial screening is conducted by a member of the research team. Eligible participants receive a detailed explanation of the study over the phone, along with an information sheet sent via email. Participants are not blind to the intervention nor the different arms. If needed, a second phone consultation is provided before obtaining informed consent. The consent form can be signed either digitally (via email) or on paper (via postal mail). An example of the informed consent form can be found in online supplemental appendix A.

### Allocation and randomisation

The randomisation process will be conducted using a Python script developed internally by a member of the research group, who is not involved in the recruitment, intervention, or analysis phases of the project. Participants will be randomised to one of the three treatment arms using the method of randomly permuted blocks with random block sizes (3–6). To maintain balance between intervention groups, randomisation will be stratified by recruitment setting (University Hospital Leuven/Antwerp University Hospital/general practitioners/self-referral). The script’s output will be transferred to a structured Excel file, which facilitates the allocation of each participant to their respective treatment arm while considering predefined strata (n=4) and assigns a unique study-specific identifier to each participant. Access to and utilisation of this Excel file will be restricted to the principal investigator and an independent member of the research group. This independent member is responsible only for allocating new participants to the treatment arms and assigning them study-specific identifiers, with no further involvement in the project.

### Interventions

#### Arm 1: eHealth self-management support programme

This programme consists of two parts. First, participants will complete a PSE programme. We assume that when participants finish the educational programme, they will be able to apply the learnt information and will be motivated to be more physically active despite barriers such as pain and fatigue. However, there is often an intention-behaviour gap. For that reason, the second part of the self-management programme consists of a goal and self-regulation programme based on the HAPA to promote an active lifestyle. Combining both PSE and PA behaviour change approaches, the key features of the eHealth self-management support programme under investigation in this project include:

- Accessibility from any digital device (laptop, tablet, smartphone) after receiving a personal login code provided by the researcher. The eHealth programme is accessible for the whole duration (ie, 1 year) of the study.
- After going through an instruction page, participants go through the eHealth programme independently at their own pace. It is recommended to do the PSE part in 6 weeks and the PA coaching in 6 weeks leading to a recommended total duration of 12 weeks.
- A chatbot goes into a conversation with the participant about persistent pain symptoms and PA. Based on targeted questions (by means of an underlying decision-tree-based algorithm created in the development phase of the project and informed by expert knowledge^45^), automated and personalised information is given about pain mechanisms and pain self-management tips. Short videos, images, external website links, reflection questions and quizzes are incorporated to address various learning styles and enhance engagement throughout the programme.
- Twenty-five different modules (see online supplemental appendix B) on different topics related to persistent pain after cancer treatment are presented according to the responses of the participants to questions within the chatbot. This way, there is a logical flow through the modules without unnecessary information being provided (eg, information about pain after chemotherapy, if the survivor has reported having no chemotherapy). The different modules are also freely accessible at all times in the main menu of the programme, when completed. Each module takes 5–30 min. The goal is to go through all information (±3 hours) over a maximum of 6 weeks. The feasibility and acceptability of this educational part of the eHealth programme were demonstrated in a pilot study by De Groef *et al*.^45^
- As part of promoting PA, after receiving the relevant PSE, participants go through a goal and self-regulation programme based on the HAPA model to promote PA.^46^,^48^ The programme consists of a daily cycle: each morning, participants are automatically directed towards the ‘goal’ module (‘goal setting’), in which they are requested to set a specific step or activity goal (‘action planning’), considering the anticipated possibilities or obstacles of that day (‘coping planning’). They have the possibility to select or formulate specific actions within four domains (transport, household, work/school, leisure time). Participants formulate the degree of difficulty of performing the action plan and how to cope with these difficulties. In the evening, the users are asked to reflect on their daily action plan by writing down barriers and facilitators (‘review behavioural goal’, ‘discrepancy between current behaviour and goal’). Participants can monitor their progress towards their daily goal (‘self-monitoring’) by using a step counter (watch or smartphone). This segment of the eHealth programme consists of three phases, each lasting 5 to 7 days, followed by a fourth phase that remains open and can be used on request. Throughout these four phases, the use of the full goal and self-regulation cycle fades out, prompting skills learning and programme-independent use of self-regulation techniques. This part of the eHealth programme is based on the eHealth programme MyDayPlan developed and tested before by the research team.^49^,^51^ This MyDayPlan intervention, based on the HAPA model, has already been found to be effective in altering health behaviours such as PA and dietary behaviour in diverse populations and diverse conditions, including chronic conditions.^49^,^51^
- The programme is a stand-alone programme that a patient can use independently. To improve outreach and sustained use of the intervention, reminders by email are provided in the first three phases.
- As part of the eHealth programme, participants can be advised to consult the healthcare provider that recruited the patient (eg,general practitioner, oncologist, anaesthesiologist). To maintain the pragmatic nature of the trial, the healthcare provider decides whether an additional consultation is needed for the particular patient (individually tailored).

#### Arm 2: Usual care

Currently, in Belgium, the standard care provided to breast cancer survivors experiencing persistent pain primarily involves a pharmacological approach and general advice regarding PA, lifestyle and psychological support.^52^ The hospitals typically distribute general brochures containing information on the long-term side effects of cancer treatments, alongside specific brochures advising individuals on how to manage pain and suggesting consultation with healthcare providers when necessary. To streamline this information, a study-specific brochure has been created to summarise available resources. Participants in this trial arm receive this study-specific brochure sent by postal mail. Consequently, participants allocated to the usual care group will not engage in any formal PSE and/or PA programmes with a physical therapist as part of this project. Although there are no restrictions placed on their ability to pursue self-initiated care or engage in PA.

#### Arm 3: Face-to-face self-management support programme

The face-to-face self-management support programme consists of the same two parts as the eHealth intervention: a PSE module, combined with an active behavioural approach, that is, a PA coaching module (24–27). The programme includes nine individual face-to-face sessions over the course of 12 weeks, each lasting 30 min, in line with the regulations of the Belgian Healthcare system. The intervention is delivered by a physical therapist in primary care who has been recruited and trained by the research team.

- In the first session, a biopsychosocial assessment is performed, and an introduction to the PSE provided in sessions two and three is given.
- The content of the PSE programme in sessions two and three is the same as for the eHealth programme. Like the eHealth programme, this education intervention includes advice for activity management while experiencing pain and other symptoms, in order to remove barriers for an active lifestyle. The educational information is presented both verbally (explanation by the therapist) and in written form (a brochure with summaries, pictures, metaphors and diagrams).
- In the fourth session, the physical therapist discusses the participant’s current behaviour regarding an active lifestyle. Principles such as graded activity or activity pacing will be discussed based on necessity.
- The fifth session focuses on setting appropriate goals for achieving an adaptive active lifestyle.
- During sessions six through nine, the physical therapist coaches and empowers the participant by reflecting on goals, discussing barriers and facilitators, setting new goals and revising concepts discussed in education as necessary.

All physical therapy sessions are reimbursed by the health insurance of the participant. The personal share of the participant will be reimbursed by the project.

### Outcomes

Assessments will be performed at baseline, after 6 weeks, 12 weeks and 6 and 12 months after baseline. However, because of feasibility limitations and relevance, not all outcome parameters are assessed at each assessment time point. Online supplemental appendix C presents a table with the study outcome measures by assessment time point. Study data will be collected and managed using Research Electronic Data Capture (REDCap) electronic data capture tools hosted at Antwerp University. REDCap is a secure, web-based software platform designed to support data capture for research studies.^53 54^

The primary outcome (primary objective) is pain-related functioning at 6 months post-baseline, assessed using the Pain Disability Index (PDI).^55^ The reliability of the Dutch version is good in the breast cancer population.^56^

As a secondary objective, this research project will determine the effect of the educational part alone of the eHealth self-management programme on pain beliefs and attitudes (key secondary outcome) at 6 weeks (ie, before the start of PA coaching). To determine other *secondary outcomes*, we formulated a programme theory delineating how intervention components, mechanisms and contextual factors of the eHealth programme contribute to intermediate, short-term and long-term results. Employing HAPA as our theoretical framework, we initiated the process by identifying the targeted behaviour (eg, an active lifestyle) and primary outcome (eg, pain-related disability). Subsequently, we identified the necessary steps in the programme and listed the used behaviour change techniques in each of these steps. We also considered contextual factors and possible factors that might hinder or facilitate (moderators) the hypothesised causal chain of mechanisms of change (mediators). Three sessions with health psychologists, physical therapists and behaviour change experts to ensure a thorough development of our programme theory. A mapping of the items of self-reported measures onto constructs relevant to the intervention took place. This allowed us to refine available items, develop new items that were relevant but missing and drop (subscales of) self-reported measures that proved irrelevant according to our programme theory. This approach ensured a comprehensive assessment of patient-related, pain-related, physical and psychosocial outcomes of our programme theory components and outcomes.

For the health-economic analysis, a self-composed questionnaire will be used to determine whether participants are working, working part-time/full-time or working in an adapted work environment. Socioeconomic outcomes will be evaluated using: (1) the Medical Consumption Questionnaire,^57^ a generic instrument for measuring direct medical costs of a patients’ total medical consumption, including additional diagnostics, consultations, surgery including stay in hospitals, physiotherapy, medication and aids prescribed by the general practitioner as well as medication and aids purchased by the patients themselves, and (2) the Productivity Cost Questionnaire^58^ to obtain data regarding the indirect costs outside healthcare, but related to the disease (eg, the costs due to absence of work and possible decreased productivity at a paid job or at an unpaid job). These two questionnaires are understandable, easy to use and generate valid data.^59 60^ The combination of these questionnaires is advised by the Institute for Medical Technology Assessment, Erasmus University Rotterdam (the Netherlands)^61^ and has served in a large number of high-quality cost-effectiveness trials. In addition, a generic health-related quality of life questionnaire, the EuroQol 5-dimension 5-level (EQ-5D-5L) questionnaire, will be administered. With this information, the incremental cost-effectiveness ratio will be calculated as general outcome of economic benefit (cost-effectiveness) and quality-adjusted life-years as outcome of enhanced quality of life (cost-utility analysis).

Parallel to the effectiveness trial, a *process evaluation* will be performed to assess (a) implementation fidelity of the intervention across the three arms, (b) the contextual factors affecting implementation and outcomes, and (c) the determinants of scalability. The design is based on the Medical Research Council guidelines for developing and evaluating complex interventions.^62^ Implementation fidelity will be assessed by measuring delivery, dosage and reach of the intervention (see online supplemental appendix D). Contextual factors affecting implementation and outcomes will be assessed at the nanolevel (patient-health care provider interaction), microlevel (individual healthcare organisation) and mesolevel (collaborative structures for transmural care) through semistructured interviews with patients, healthcare providers and key informant interviews. Scalability of the intervention is considered through its feasibility of integration into routine healthcare processes. Data will be collected through observations and interviews with the key informants and stakeholders at the healthcare organisation level and policy level. These observations and interviews will be organised during and shortly after the intervention.

### Sample size

This study adopts a three-arm design to evaluate the eHealth intervention’s superiority to usual care and non-inferiority to face-to-face intervention using a hierarchical testing procedure.^63^ The trial will be termed successful if superiority of the eHealth intervention to usual care as well as non-inferiority of the eHealth intervention to the face-to-face intervention has been shown. A hierarchical testing procedure will be applied, first testing superiority, followed by testing non-inferiority if and only if superiority was shown. Consequently, no correction for multiple testing needs to be applied. Note that if superiority was shown, but showing non-inferiority failed, it is still allowed to conclude that eHealth intervention is effective, but not that it can replace the face-to-face intervention.

The primary outcome used to test superiority as well as non-inferiority is the PDI measured at 6 months. Both tests will be based on the comparison of the mean value after correction for the baseline PDI (Analysis of Covariance (ANCOVA) approach). The assumed SD of the PDI and the correlation between baseline and 6 months equal 15 and 0.65, respectively, and were obtained from pilot data on 28 subjects.^45^ No difference in PDI was expected between face-to-face and eHealth self-management and the region of non-inferiority was defined as a difference of five PDI points. The sample size was calculated to have at least 80% power to show non-inferiority based on a one-sided test with alpha equal to 0.025 (hence, equivalent to a two-sided test with alpha=0.05). A simulation study with 2000 simulations was used, and the power was defined as the percentage of sampled datasets where non-inferiority has been shown using the ANCOVA approach. With 79 subjects in the eHealth and the face-to-face group, there is at least 80% power to show non-inferiority.

To show superiority of eHealth intervention vs usual care based on a two-sided (ANCOVA) test with alpha equal to 0.05, 83 subjects per group are required to detect with at least 80% power a difference of 5 points. Note that having at least 80% power for each of the two comparisons separately does not guarantee 80% power simultaneously.

Strictly speaking, fewer patients are required in the face-to-face group compared with the two other groups. However, given the small difference (79 vs 83), it was decided to recruit an equal number of patients in each of the three groups.

Based on the number of subjects required for the primary outcome, more than 80% power is also guaranteed for the key-secondary outcome Survey of Pain Attitudes (SOPA). Indeed, to detect a difference in SOPA of 4 points, assuming an SD of 7.5 and a correlation between baseline and 6 months of 0.30, 52 subjects per group are required. Further, 50 subjects per group are required to show with at least 80% power non-inferiority of eHealth intervention, setting the margin of non-inferiority equal to 4 points. Assumptions for the key secondary outcome measure are based on the pilot study of Moseley *et al* on PSE in a non-cancer population.^64^

To anticipate a dropout percentage of 7%, the sample size in each group will be increased with factor 100/93=1.075, yielding 90 subjects per group, hence 270 subjects in total.

### Data analysis

A constrained longitudinal data analysis model (cLDA) will be used to compare the PDI between the three groups.^65^ In the cLDA, both the baseline and post-baseline values (12 weeks, 6 months and 12 months) are modelled as dependent variables, as opposed to a (longitudinal) ANCOVA model in which the baseline value is included as a covariate. Although the baseline measure is included in the response vector in cLDA, the true baseline means are constrained to be the same for different treatment groups due to randomisation, and this analysis provides an adjustment for the observed baseline difference in estimating the treatment effects. As opposed to the ANCOVA model, the cLDA approach can handle the presence of missing data and produces unbiased estimates if the missing data mechanism is missing at random. Additionally, 95% CIs for the group-specific mean post-baseline values will be constructed as well as the confidence intervals for the differences between the groups. The stratification variable will be added as a fixed factor in the model.

The primary analysis refers to the comparison after 6 months. A hierarchical testing procedure will be applied for the two primary comparisons. First, the difference between the eHealth intervention and usual care will be tested based on a two-sided test with alpha set at 0.05. If significant, non-inferiority of the eHealth intervention vs the face-to-face intervention will be tested based on a one-sided non-inferiority test with alpha set at 0.025 and defining the margin of non-inferiority as a difference of 5 PDI points. For the superiority comparison, the analysis will be based on the intention-to-treat principle, that is, all randomised patients will be analysed in the allocated group. However, for the non-inferiority comparison, the analysis will be based on the as-treated analysis set.

For the continuous secondary outcomes, the same cLDA model as for the primary outcome will be used. For the key-secondary outcome SOPA, a non-inferiority test (eHealth vs face-to-face intervention) will be performed defining the margin of non-inferiority as a difference of 4 points (one-sided test with alpha equal to 0.025). Note that for the other secondary outcomes, no inferiority tests will be performed, but all differences between groups will be evaluated using two-sided 95% CIs. The alpha-level for the secondary outcomes is set at 0.05, without a priori planned corrections for multiple testing. Thus, only for the key-secondary outcome SOPA a strong claim is possible without correction for multiple testing. Data analysis will be performed by The Leuven Biostatistics and Statistical Bioinformatics Centre of KU Leuven, who is blinded to group allocation.

### Data security and management

Participant data will be stored on a secure database (REDCap) in accordance with the General Data Protection Regulations (2018). Data are de-identified and a unique trial identification number used on all participant communication. Clinical and patient forms will be checked for completeness and congruity before data entry onto the database. Data will undergo additional checks to ensure consistency between data submitted and original paper forms. Trial documentation and data will be archived for at least 10 years after completion of the trial.

### Trial monitoring

The trial steering committee will oversee all aspects of design, delivery, quality assurance and data analyses. The trial steering committee does not have direct access to the data, but may have access to reports on data quality, for example. The data monitoring committee will check completeness, validity and data quality and report to the trial steering committee about this.

## Ethics and dissemination

### Ethical considerations

The PECAN trial applies the principles established in the Declaration of Helsinki.^66^ Participants provide written informed consent before data collection. Only de-identified coded and interpreted data will be shared between the members of the research team. Ethical approval was granted by the Ethical Committee of the University Hospitals Leuven and Antwerp: BUN B3002023000132

### Dissemination of results

The research team is committed to full disclosure of the results of the trial. Findings will be reported following CONSORT guidelines, and we aim to publish in high-impact journals. The funder will take no role in the analysis or interpretation of trial results.

## Discussion

This protocol describes a pragmatic, three-arm randomised controlled trial evaluating the effectiveness of an innovative eHealth self-management support programme for persistent pain after breast cancer treatment. By comparing the eHealth intervention to both usual care and a face-to-face programme, the study aims to assess its superiority and non-inferiority, respectively. The intervention is grounded in a biopsychosocial model and leverages PSE and behaviour change principles to promote an active lifestyle. Delivered via a chatbot, the programme is designed to be accessible, scalable and cost-effective. Its digital format offers the potential to overcome common barriers to pain rehabilitation, such as limited availability of specialised care and time constraints. The trial is conducted in both hospital and primary care settings to enhance external validity. Pain-related disability at 6 months is the primary outcome, supported by a broad set of secondary outcomes assessing physical, psychological and health-economic effects. Patient input informed the design, ensuring relevance and feasibility. Results from this trial will contribute valuable evidence on the role of eHealth self-management in cancer-related pain rehabilitation and inform future implementation strategies.

## Supplementary material

10.1136/bmjopen-2025-099241 online supplemental file 1

10.1136/bmjopen-2025-099241 online supplemental file 2

10.1136/bmjopen-2025-099241 online supplemental file 3

10.1136/bmjopen-2025-099241 online supplemental file 4
