# Supplementary material for: Study protocol for a pragmatic randomised controlled trial in Belgian primary care and hospital settings on the effectiveness of an eHealth self-management support programme consisting of pain education and coaching of activity needs in breast cancer survivors with persistent pain: the PECAN trial
Source: BMJ Open. 2025 Aug 22;15(8):e099241. doi: 10.1136/bmjopen-2025-099241 (PMC12374636; doi:10.1136/bmjopen-2025-099241)
Supplement: online supplemental file 1 [file bmjopen-15-8-s001.pdf]

INFORMATION DOCUMENT FOR THE PARTICIPANT IN A NON-COMMERCIAL EXPERIMENT ON THE HUMAN PERSON

Title of the study:
Effectiveness of self-management programs for persistent pain after breast cancer treatment

Sponsor:
University of Antwerp - Faculty of Rehabilitation Sciences and Physiotherapy: MOVANT research group
[redacted]

Medical Ethics Committee:
UZA-UA Ethics Committee
[redacted]

Ethics Committee UZ Leuven – EC research
[redacted]

Dear participant,

You are invited to **voluntarily** participate in a study that investigates the effect of three different self-management programs for persistent pain after breast cancer. Before you agree to participate in this study, it is important that you read this letter.

This study was approved by the independent Ethics Committee of the University of Antwerp on 30-01-2024

Purpose of the study

About 1 in 2 women experience **persistent pain after** breast cancer treatment. Surgery for breast cancer, but also radiotherapy, chemotherapy and/or hormone therapy can cause pain. However, this pain can also remain present (long) after these treatments or only arise. When pain complaints are present for more than 3 months, these are called persistent pain complaints. Persistent pain symptoms can severely limit a person's functioning in daily life (e.g. household, work, sports) and **can lead to a generally reduced quality of life**.

At the moment, persistent pain complaints are mainly addressed with medication. However, this approach doesn't work for everyone, and many individuals experience unpleasant side effects. However, there are other non-pharmacological options for dealing with persistent pain.

For example, there is already scientific evidence that **pain education** and adopting an **active lifestyle** works for persistent pain complaints of, for example, the back. Pain education is explaining what pain is, how pain arises and how a person can deal with (persistent) pain complaints. In current practice, pain education and physical activity support are provided face-to-face by physiotherapists in a hospital or primary care practice. However, this approach requires significant (financial) resources and can be hampered if patients themselves have limited resources, mobility, motivation or time due to e.g. their work schedule. In addition, patients are often already burdened with long-term follow-up of medical supervision and hospitals can be stressful.

However, scientific research also shows that providing pain education and encouraging people to adopt an active lifestyle can also be done through **self-management programs**. This is a form of therapy in which the patient's independence and independence are central. This means that people decide for themselves about the care and support ('doing it themselves/managing').

The aim of this study is to investigate the effect of **3 different self-management programs** on pain-related functioning in individuals with persistent pain after treatment for breast cancer:

1. **eHealth self-management**: self-management using a digital platform (eHealth are digital applications in healthcare for e.g. communication, evaluation and treatments)
2. **Face-to-face self-management**: self-management under the guidance of a physiotherapist
3. **Self-management brochure**: self-management based on written information

Inclusion criteria for participants

The following individuals (men and women) are eligible to participate in this study:

- Treated for a primary breast cancer through surgery. 'Primary' refers to the original, first place where a cancer arose in the body, in this case in the breast.
- Primary treatment for breast cancer must be completed at least 3 months prior to study entry. Treatment with hormonal therapy and/or immunotherapy is allowed at the time of participation.
- Persistent pain for 3 months that affects daily activities.
- Speaking and understanding the Dutch language.
- Participation possible during the entire study period.
- Have not previously participated in an education program for persistent pain.

Conduct of the investigation

After a baseline evaluation, participants will be randomly assigned to 1 of 3 different groups. An interim evaluation will take place in 6 weeks. After 12 weeks, the intervention will be completed and another evaluation will take place. This evaluation will be repeated at 6 and 12 months after the baseline evaluation.

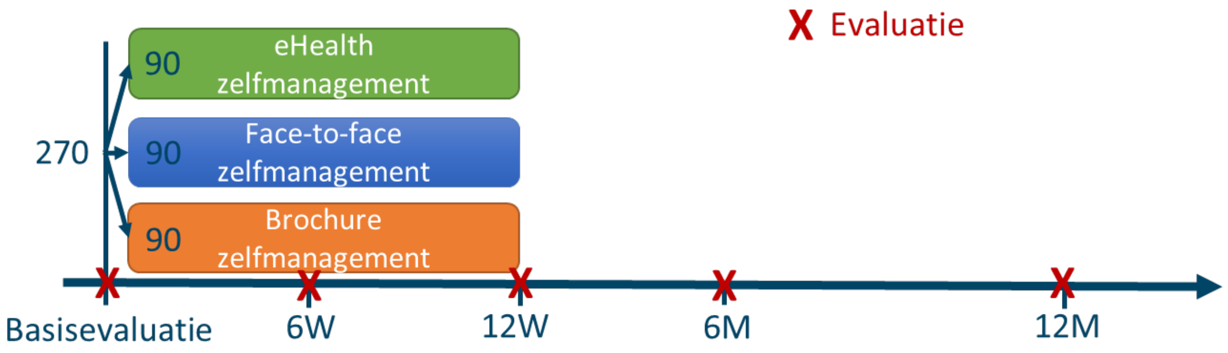

<u>Intervention</u>

eHealth self-management

Participants will independently go through a digital program that can be accessed from any digital device (laptop, tablet, smartphone). The investigator will provide the participant with a personal login code for the program. The program consists of two parts. In the first part, relevant information about pain after breast cancer is given on the basis of short conversations with a chatbot. This information prepares the participant for the second part of the self-management program, which consists of daily activity planning and strategies to promote an active lifestyle. Participants go through a cycle every day in which self-regulation techniques are offered. The cycle consists of several steps: each morning, participants are asked to set a specific step goal, taking into account the expected opportunities or obstacles of that day. Participants should indicate how difficult it is to implement the action plan and how they can deal with these difficulties. In the evening, the participant is asked to reflect on what they have done that day of activities. Participants will be able to track their own goals using a pedometer of their choice (e.g., Fitbit, Garmin, iWatch, their own mobile phone; their own or borrowed from the investigators).

Face-to-face self-management

Participants will be referred to a physiotherapist near their place of residence. First, 3 individual face-to-face sessions of pain education will be received from the physiotherapist. The information is presented orally (explanation by the therapist) and in writing (information leaflet, images). After the education, the physiotherapist discusses with the patient how to set the right

goals to promote an active lifestyle. To achieve this goal, supervised sessions can be organized in groups or alone based on the patient's needs. The supervised sessions are combined with physical activities at home.

Self-management brochure

Participants will receive information about the (long-term) side effects of treatment for cancer and what to do in case of pain with the recommendations to consult a healthcare provider. This information is given to the participant by means of a brochure that is sent by post.

Evaluation

If you agree to participate in this study and if you meet all the conditions for participation, your pain-related, physical, psychosocial and work-related functioning will be evaluated on the basis of questionnaires via an online platform. The evaluation moments take place before the start of the study, as well as at 6 weeks, 12 weeks, 6 and 12 months after the start of the study. Below you will find an overview of the different questionnaires, the content and duration to complete them. You can view the questionnaires before you decide to participate in the study. If desired, the questionnaires can also be completed in writing.

Table 1

Questionnaire	Expensive	Description
Demographic data	4 minutes	Here, demographic issues such as age, gender, weight, ethnicity, education level, health and digital literacy, other physical complaints, medication use are questioned
Pain Disability Index	3 minutes	Evaluates pain-related impairment
Margolis Pain diagram	1 minute	Evaluates localization of pain
Neuropathic Pain Symptom Inventory	3 minutes	Evaluates presence of neuropathic pain
Neurophysiology of pain questionnaire	8 minutes	Evaluates knowledge about the neurophysiology of pain
PROMIS-57 Profile v2.01	10 minutes	Evaluates physical functioning, fatigue, sleep, anxiety, depression and participation, well-being
PROMIS-57 Profile v2.04a	5 minutes	Evaluates the experience of social support
Patterns of Activity Measure	4 minutes	Evaluates different activity patterns
Survey Of Pain Attitudes<br><br>(only at the start and after 6 weeks)	6 minutes	Evaluates attitudes and thoughts about pain

Pain Catastrophizing Scale	2 minutes	Evaluates catastrophizing over pain
Bodily Threat Monitoring Scale	5 minutes	Evaluates alertness to bodily sensations
Determinants of physical activity	4 minutes	Evaluates different determinants of physical activity
PROMIS Global Health	2 minutes	Evaluates health-related quality of life (for measuring the effect of the intervention)
EQ-5D-5L	2 minutes	Evaluates health-related quality of life (for the health economic analysis)
Productivity Cost Questionnaire	10 minutes	Evaluates employment status (for the health economic analysis)
Medical Consumption Questionnaire	20 minutes	Evaluates health-related costs (for the health economic analysis)
Patient Global Impression of Change	2 minutes	Evaluates the perceived effect of the intervention

In addition to these questionnaires, each participant will be asked to wear an activity meter at each evaluation moment for 7 days (min. 12 hours per day for 7 days). This meter measures when and how much you are physically active in daily life. This meter will be sent to you by post and can also be returned (free of charge) to the research team after 7 days.

Duration of the study

This study consists of 4 evaluation moments (baseline evaluation, 12 weeks, 6 and 12 months after the baseline evaluation) that will each take up about 80 minutes of your time. The interim evaluation at 6 weeks is shorter and will take about 6 minutes of your time. The evaluation is done entirely online, so no additional travel is necessary. The duration of the self-management program is 12 weeks.

In the future, research may follow that is in line with this study. At the last evaluation moment, you will be asked to leave your contact details without obligation, so that we can contact you in connection with other future studies.

Pros and Cons

The benefit of this study is following a self-management program around persistent pain after breast cancer, which will help you gain more knowledge on this topic. We cannot guarantee that if you agree to participate in this study, you will personally receive any direct benefit from your participation in this study. However, the information provided by this study can contribute to a better knowledge of the treatment of patients with persistent pain after breast cancer. Your participation in this study does not pose any health risk. Participation in the study could possibly be a confrontation with some new themes about mental, physical and social well-being. If you wish, you can always contact a researcher for a supportive meeting. Deciding not to participate in the study will also have no consequences. You also have the right to stop participating in the study at any time, even after you have signed the informed consent form.

1. General information about participating in a study

Voluntary participation

If you agree to participate in the study, you should keep this information leaflet and you will be asked to sign the attached consent form.

You participate in this study entirely voluntarily and you have the right to refuse to participate in it.

You also have the right to stop participating in the study at any time, even after you have signed the informed consent form. You do not have to state a reason for this. Withdrawing your consent will not result in any disadvantage or loss of benefits.

Your participation in the study may also be terminated at any time by the researcher(s), the Ethics Committee or the sponsor without your consent. Possible reasons for such a decision may include:

- You do not comply with the instructions for participation in the study
- Your continued participation appears to be harmful to you
- During the examination, it is determined that you do not or no longer meet the conditions for participation

Liability and insurance

If you suffer damage as a result of your participation in this study, you or your successors will be compensated for this damage by the sponsor of this study, in accordance with the applicable Belgian legislation. You do not need to prove an error for this.

The client has taken out a flawless insurance policy that covers any damage that would result from this investigation. You or your beneficiaries can summon this insurer directly at any time.

Costs and reimbursement

This study will not entail any additional costs for you. All costs arising from your participation in the study are the responsibility of the investigator or the sponsor. You will not be compensated for your participation in this study.

Protection of your privacy

Your identity and your participation in this study will be treated as strictly confidential. You will not be identified by name or in any other way in any files, results or publications related to the study.

In order to guarantee your privacy with regard to the storage and processing of the data in the context of this study, your data will be pseudonymised. This means that your surname, first name, date of birth will be replaced by a code. All further processing is carried out on this pseudonymised data. The link between the code and your person is preserved. This link is only used to provide you with certain information in your own interest.

Protection of your personal data

If you agree to participate in this study, this means that you consent to the use of your personal data collected in the context of this study.

You can withdraw your consent to the collection and processing of your data at any time. If your study participation is stopped prematurely, your original consent will allow the use of the data collected about you for the period you were involved in the study.

Only persons directly involved in the research will have access to your personal data. Your data will not be passed on to third parties.

The researchers will keep your data for a period of 25 years.

You have the right to ask the researcher what data is collected about you in the context of the research and what its purpose is. You can ask for certain data to be corrected or deleted, or for your data to no longer be used.
All data collected from you will be treated in accordance with the "Directive on the Protection of Individuals with regard to the Processing of Personal Data" and the national legislation applicable to it. The European General Data Protection Regulation (GDPR) – EU2016/679) and the Belgian legislation that further elaborates this regulation.

As the sponsor of the study, the University of Antwerp is responsible for the processing of your personal data. To this end, it has appointed a data processing officer.

Questions regarding the management of your data can be directed to the researchers, to the data protection officer of the University of Antwerp or KU Leuven via:

DPO University of Antwerp:
Campus Middelheim, Middelheimlaan 1, 2020 Antwerp, privacy@uantwerpen.be
[redacted]
More info: https://www.uantwerpen.be/nl/overuantwerpen/missie-en-visie/privacyverklaring/
DPO KULeuven:
Krakenstraat 3 bus 5516, 3000 Leuven (legal department), dpo@kuleuven.be
[redacted]
More info: https://admin.kuleuven.be/privacy/index

If you feel that your rights with regard to your personal data are not sufficiently respected, you can always contact the data protection officer (see above) who will take the necessary measures if necessary. You also have the right to lodge a complaint with the Belgian Data Protection Authority.

We hope that this document has given you sufficient information about the research.

You have the right to ask additional questions at any time about the content, purpose or course of the research, about the possible and/or known advantages and disadvantages that this research entails for you, etc.. You can contact the researcher(s) *(and the contact person mentioned above) for this*.

CONSENT FORM

Part only intended for the participant:

I, the undersigned, hereby confirm .... *(name and first name of participant* that I have been informed about the study and have received a copy of the information document.

I have read and understood the information. The researcher has given me sufficient information regarding the purpose and design of the study, its conditions and duration, and the possible known advantages or disadvantages that this study may entail for me. In addition, I was given sufficient time to consider the information and to ask questions, to which I received satisfactory answers.

I am aware that I can refuse my consent to participate in the study and to process my data and that I can withdraw the consent to use my data at any time, after informing the researcher, without causing me any harm. My original consent to participate in the study and use of my personal data will allow the use of my data with respect to the period I was involved in the study.

I am aware of the purpose for which my data is collected, processed and used in the context of this research. I have been informed that my data will be kept for 25 years for scientific research.

I know that I have the right to access and correct my data. If I have any complaints about the management of my data, I know that I can contact the researchers or the data protection officer of *the University of Antwerp* or KU Leuven via:

DPO University of Antwerp:
Campus Middelheim, Middelheimlaan 1, 2020 Antwerp, privacy@uantwerpen.be
[redacted]
More info: https://www.uantwerpen.be/nl/overuantwerpen/missie-en-visie/privacyverklaring/
DPO KULeuven:
Krakenstraat 3 bus 5516, 3000 Leuven (legal department), dpo@kuleuven.be
[redacted]
More info: https://admin.kuleuven.be/privacy/index

I give permission to the researcher to collect, process and use my data as described in the information document I received and in accordance with the applicable legislation. My data will be treated in strict confidence. When the study results are published, my identity will remain secret.

I voluntarily agree to participate in this research and to cooperate in all requested research actions (*going through a self-management program and filling in questionnaires).*

I agree that researchers with access to this database may contact me in the future about possible participation in other studies. I understand that I can have my data removed from the database at any time by contacting a researcher affiliated with the oncological rehabilitation research group.

I agree that the following data will be stored (circle what applies):

Surname	**Yes / No**
First name	**Yes / No**
Address	**Yes / No**
Telephone number	**Yes / No**
Email address	**Yes / No**
Date of birth	**Yes / No**
Name of my treating GP	**Yes / No**
Name of my treating oncologist	**Yes / No**
Disease severity (TNM stage)	**Yes / No**
Received/planned treatment (surgery, chemotherapy, radiotherapy, hormone therapy, immunotherapy)	**Yes / No**

I have read and approved the above.

_____________________ _____________ _____________________

Name of the participant Signature Date *(day/month/year)*
(capital letters)

Part intended for the physician-researcher only:

I, the undersigned ...., authorized research officer, declare that I have provided the necessary information regarding this study orally and that I have provided a copy of the information document to the participant.

I confirm that no pressure has been exerted on the participant to get him/her to agree to participate in the study and I am willing to answer any additional questions you may have.

I confirm that I work in accordance with the ethical principles as stated in the latest version of the "Declaration of Helsinki", the "Good Clinical Practice" and the Belgian law of 7 May 2004 on experimentation on the human person.

______________________________	_______________	__________________________
Name of the physician-investigator *(capital letters)*	Signature	Date *(day/month/year)*
