# Supplementary material for: Study protocol for a pragmatic randomised controlled trial in Belgian primary care and hospital settings on the effectiveness of an eHealth self-management support programme consisting of pain education and coaching of activity needs in breast cancer survivors with persistent pain: the PECAN trial
Source: BMJ Open. 2025 Aug 22;15(8):e099241. doi: 10.1136/bmjopen-2025-099241 (PMC12374636; doi:10.1136/bmjopen-2025-099241)
Supplement: online supplemental file 2 [file bmjopen-15-8-s002.docx]

*Overview of the content of the 25 sessions of the eHealth pain science education program*

| **Title** | **Target concepts/content** |
| --- | --- |
| 1. Introduction | - Background about who developed the program and the evidence underpinning the program - Outline and goal of the program - Introduction on self-directed learning - Reflection on the motivations for undertaking the program - Preliminary goal setting |
| 2. Learn how you learn | - Reflection on the learning style of the user - Strategies to optimize learning |
| 3. Boost your learning | - How to apply new information to one’s personal situation - Stimulation of curiosity in learning about pain - Quizzes to learn about different ways of learning, to find the best way to retain information for that specific person |
| 4. Learning about pain can influence your pain | - Why learning about pain can be helpful - How learning about pain can be helpful |
| 5. The purpose of pain is protection | - The purpose of pain is protection from potential bodily danger - Pain is not a reflection of tissue damage - An introduction to the possible influences on the perception of pain |
| 6. Time heals all wounds | - The brain can produce pain without tissue damage - Tissues heal in days/weeks and the different stages of normal the tissue healing - It is now safe and beneficial to move |
| 7. Scars as a sign of perseverance | - Scars are stories of strength and survival - How to self-manage and soften scars |
| 8. Without brain, no pain | - The brain makes pain, and you can’t feel pain without it - The brain has to take multiple factors into account, before we can talk about pain - The brain works as a danger alarm system, with pain as its siren |
| 9. About alarm signals, damaged nerves and sensitisation | - Altered danger alarm system when pain persists - What does sensitisation entail and examples of sensitisation, as well as its reversibility - Mechanisms behind neuropathic pain and related unusual sensations |
| 10. How do nerves heal? Slow but steady | - What is neuropathic pain and neuropathies and do I experience it? - How nerves move and work and what influences them - Recovery time of neuropathies - Strategies to self-manage neuropathies |
| 11. You cannot stop bioplasticity | - What is bioplasticity, how does it affect our bodies and what influences bioplasticity - The inevitability of the body’s innate ability to heal and learn - Implications of the pain system learning/adapting to recovery |
| 12. Multiple influences on pain | - Pain can be modified by anything - Introduction of Danger in Me (DIMs) and Safety in Me (SIMs) factors |
| 13. In the shadow of the volcano | - Living with pain-related uncertainty - The pitfalls of over-monitoring - Strategies to deal with this pain-related uncertainty |
| 14. The immune system | - The effect of the immune system on pain - Relating immune effects to pain symptoms and behaviors |
| 15. From radiation to rehabilitation (*optional)* | - Biopsychosocial mechanisms behind side effects of radiotherapy - Skin effects of radiotherapy - Skin care following radiotherapy - Flare-ups without re-injury |
| 16. Chemotherapy also influences the nerves *(optional)* | - Biopsychosocial mechanisms behind side effects of chemotherapy - Helpful and unhelpful strategies for self-management of chemotherapy-related signs and symptoms |
| 17. Don’t blame the hormones *(optional)* | - Biopsychosocial mechanisms behind hormone therapy and the related signs and symptoms - Role of the endocrine system in pain - Importance of movement, sleep management, diet and SIM’s in management of hormone-therapy related signs and symptoms |
| 18. Train the brain | - What do you need to do to retrain your pain system to be less protective again |
| 19. Use it or lose it | - Impact of breast cancer treatments and inactivity on the body’s tolerance for exercise - Signs and symptoms that people might experience when resuming exercise - General tips for becoming more active |
| 20. Recharge your batteries | - Think about what you are already doing or not doing - Graded activity principles - The importance of pacing and planning your activities and breaks - Tips and resources for setting goals - Idea of energy-givers and energy-takers |
| 21.Exercise as medicine | - Why should I have an active lifestyle? - Recommendations regarding physical activity and sedentary behaviour - Self-management advice on how to get or improve your active lifestyle |
| 22. Cancer-related fatigue | - What is cancer-related fatigue and where does it come from - The importance of physical activity to combat cancer-related fatigue - Self-management strategies for cancer-related fatigue - Other resources for self-learning, reflection and management of cancer-related fatigue |
| 23. Stress less is best! | - Stress as an important protection mechanism - Stress and persistent pain are inter-related - How stress affects the body - Stress self-management strategies - Other resources for self-learning, reflection and management of stress |
| 24. Rest your brain and your pain: sleep management | - The importance of sleep for people experiencing pain - Myths about sleep and its management - Advice and strategies for good sleep hygiene practices - Other resources for self-learning, reflection and management of sleep |
| 25. Food for thought: nutrition | - Nutrition, diet and pain are inter-related - Dietary principles and optimal nutrition - Alcohol consumption, pain and breast cancer treatment |

*Overview of the 4 phases of the eHealth active lifestyle promotion program*

Phase 1: Active lifestyle promotion with full support

The objective is to learn and apply goal setting and self-regulation techniques over the number of days that they need to achieve 5 ‘good’ days (which means that they achieve their set goal) . Each day, participants set a physical activity goal for that day (goal setting), and they are supported in developing an action plan detailing how and when activities will take place to achieve that goal (action planning). They are also prompted to reflect on possible barriers to these activities and to come up with possible solutions to overcome these barriers (coping planning). Participants are instructed to keep track of their activities (self-monitoring), which they can complete on the platform. At the end of each day, participants consider the activities and reflect on their achievement to learn from these for the upcoming days (“discrepancy between current behavior and goal”, “review behavior goal”). Participants are informed about the objective of phase 1 and are guided through the different behavior change techniques with options, examples, and tips and tricks. The program provides feedback on their level of activities and their achievements over the course of 5 good days (which means that they achieve their set goal) via a dashboard. The participant receives a reminder email in the morning and the evening to complete the different steps of phase 1.

Phase 2: Active lifestyle promotion with optional support

The objective of this phase is for participants to autonomously apply the goal-setting and self-regulation cycle with optional support if needed for the next 7 consecutive days. The logic and layout are similar to phase 1. However, participants are instructed to only set a daily goal (goal setting), self-monitor (self-monitoring), and reflect on the goal progress of that day (“discrepancy between current behavior and goal”, “review behavior goal”). Action planning and coping planning are optional during these days, and participants are encouraged to use these when experiencing difficulties in achieving their daily goals. The participant receives a reminder email in the morning and the evening to complete the different steps of phase 2.

Phase 3: Active lifestyle promotion without support

The objective is for participants to autonomously apply the goal-setting and self-regulation cycle without support for the next 7 consecutive days. The logic and layout are similar to phase 1, with the exception that participants are no longer able to fill in daily goals, action, and coping plans. The idea is that these techniques have been learned, and participants can now apply them independently. There is no optional support. Participants self-monitor (self-monitoring) their activities, and at the end of each day, they reflect on the goal progress of that day (“discrepancy between current behavior and goal”, “review behavior goal”). The participant receives a reminder email in the evening to complete the different steps of phase 3.

Phase 4: Active lifestyle promotion with optional support available on request

Participants have learned to use the goal-setting and self-regulation techniques and can apply these in their daily lives. However, relapses may occur, or difficulties may be experienced. Participants can return to phase 2 for optional support at any time.
