# Supplementary material for: Study protocol for a pragmatic randomised controlled trial in Belgian primary care and hospital settings on the effectiveness of an eHealth self-management support programme consisting of pain education and coaching of activity needs in breast cancer survivors with persistent pain: the PECAN trial
Source: BMJ Open. 2025 Aug 22;15(8):e099241. doi: 10.1136/bmjopen-2025-099241 (PMC12374636; doi:10.1136/bmjopen-2025-099241)
Supplement: online supplemental file 3 [file bmjopen-15-8-s003.docx]

APPENDIX C. Study outcome measures by assessment time point.

| **Outcome** | **Questionnaire** | **Description** | **Timepoint** | | | | |
| --- | --- | --- | --- | --- | --- | --- | --- |
|  |  |  | Baseline | 6 wks after baseline | 12 wks after baseline | 6 Mo after baseline | 12 Mo after baseline |
| **Demographics – patient-related factors** | | | | | | | |
| Age, weight, height, sex, educational level, marital status, housing, family structure, socio-economic status, nationality | Self-composed | The patient demographics will be assessed with a self-composed questionnaire | X |  |  |  |  |
| Comorbidities | Self-composed |  | X |  |  |  |  |
| Stage and type of breast cancer | Self-composed | Stage, type of breast cancer and the treatment characteristics will be inquired, using a self-composed questionnaire | X |  |  |  |  |
| Cancer treatment characteristics | Self-composed |  | X |  |  |  |  |
| Health literacy | The European Health Literacy Survey-6 questions (HLS-EU-Q6) | Health literacy will be evaluated with the European Health Literacy Survey Questionnaire (HLS-EU-Q6)[1] | X |  |  |  |  |
| Digital literacy | eHealth Literacy Scale (eHEALS) | Digital literacy will be evaluated with the eHealth Literacy Scale (eHEALS)[2] | X* |  |  |  |  |
| **Pain-related outcomes** | | | | | | | |
| Pain-related disability (**primary outcome at 6 months post-baseline**) | Pain Disability Index (PDI) | Pain-related disability will be assessed with the Pain Disability Index (PDI) and is the primary outcome measure. The PDI has been tested on reliability, validity and responsiveness in Dutch in breast cancer patients by the KU Leuven research group [3]. | X |  | X | X | X |
| Pain beliefs and attitudes (**key secondary outcome)** | Survey Of Pain Attitudes Brief version (SOPA-B) | Attitudes and beliefs about pain will be assessed with the Survey of Pain Attitudes Brief version [4]. In addition 7 self-composed items on beliefs about complaints in general are added. | X | X | X | X | X |
| Pain-related worrying | Pain Catastrophizing Scale (PCS) | The Pain Catastrophizing Scale (PCS) is a 13-item questionnaire that reflects on previous painful experiences and asks to indicate the degree to which each of the 13 described thoughts or feelings were experienced while in pain (rumination about pain, magnification of negative consequences in the context of pain and experienced helplessness in the context of pain) [5]. | X |  | X | X | X |
| Knowledge of neurophysiology of pain | Study-specific version of the Neurophysiology of Pain Test | The knowledge and understanding of pain neurophysiology will be assessed with the Neurophysiology of Pain Test. The Neurophysiology of Pain Test has been translated into Dutch and the validity and test–retest reliability has been confirmed [6]. The test was adapted for this specific trial. | X | X |  |  |  |
| Pain severity: at the present moment – mean, minimum, maximum past week | Visual Analogue Scale (VAS) | Pain severity will be evaluated with a Visual Analogue Scale (VAS). Four VAS scales will be completed: pain intensity at the present moment, mean pain intensity (global average pain intensity over the past week), maximum pain intensity (pain intensity at its maximum over the past week) and minimum pain intensity (pain intensity at its minimum over the past week). | X |  | X | X | X |
| Pain location | Margolis Pain Diagram | Pain location will be evaluated with a Margolis Pain Diagram. Presence-absence of pain is checked on a body diagram for 74 predefined areas [7]. | X |  | X | X | X |
| Medication use | Self-composed | Medication use and response to medication will be assessed with a self-composed questionnaire. | X |  | X | X | X |
| Severity of neuropathic pain | Neuropathic Pain Symptom Inventory (NPSI) | The severity of neuropathic pain will be assessed with the Neuropathic Pain Symptom Inventory, a questionnaire generating reliable and valid data for identifying pain of predominantly neuropathic origin based on self-report [8]. | X |  | X | X | X |
| **Physical outcomes** | | | | | | | |
| Physical activity level | Accelerometers | General PA will be assessed with a waist-worn tri-axial accelerometer (ActiGraph wGT3X-BT11, Pensacola, FL, USA). The ActiGraph GT3X1 has demonstrated excellent relative reliability (2-week interval) for sedentary behavior and good relative reliability for moderate-to vigorous physical activity in patients 12 months after breast cancer surgery [9]. Each participant will be instructed to wear the accelerometer around the hip for seven consecutive days and for at least twelve hours per day, except during activities including water such as swimming or showering. Data collection is valid if the accelerometer is worn for at least 4 days per week with a recording of equal or more than 600 minutes. A sample rate of 90 Hz, 60-second epoch setting, and modified version of the Choi algorithm (60-0-1 using vector magnitude) will be applied. The outcomes of interest are 1) average step count, 2) time spent in different physical activity levels, 3) sedentary time. | X |  | X | X | X |
| Physical activity patterns | Patterns of Activity Measure – Pain   (short form)  Patterns of Activity Measure - Fatigue (self-composed) | The Patterns of Activity Measure-Pain Short Form (POAM-P) measures avoidance and persistence behavior [10]. The POAM-P is a 15-item self-report measure developed to identify 3 activity patterns in patients with chronic pain: avoidance (5 items), overdoing (i.e., persistence behavior; 5 items), and pacing (5 items). Participants have to indicate to which extent the item applies to them on a 5-point scale ranging from 0 (not at all) to 4 (always). Total scores per subscale range from 0 to 40. In addition to pain, fatigue can affect activity patterns. Seven self-composed items based on the POAM-P were added for this purpose (7 items). Participants fill out these additional items when fatigue is present. | X |  | X | X | X |
| Physical functioning | PROMIS-57 Profile v2.01 | The PROMIS 57 Health Profile consists of several scales that can be independently used, amongst which the PROMIS-Short Form v2.01 Fatigue scale, PROMIS-Short Form v2.01 Physical function scale, and the PROMIS-Short Form v2.01 Sleep disturbance scale.  Subscales have been validated using Item Response Theory.  Each scale consists of 8 items. Participants respond about their experience using a 5-point ordinal rating scale. The anchors vary as a function of the scale/item (e.g. Fatigue: 1-Not at all to 5-Very much; Sleep: 1-Very poor to 5-Very good; Physical function: 1-Without any difficulty to 5-Unable to do). Raw score totals are converted to an item response theory-based T-scores [11]. | X |  | X | X | X |
| Fatigue |  |  | X |  | X | X | X |
| Sleep disturbance |  |  | X |  | X | X | X |
| **Psychosocial outcomes** | | | | | | | |
| Participation | PROMIS-57 Profile v2.01 | The PROMIS Short Form Ability to participate in Social Roles and activities is part of the PROMIS-57 health profile v2.01. It consists of 8 items and assesses social role functioning and activities.  For each scale, respondents are asked how often in the past 7 days they have experienced specific depression/anxiety symptoms, using a 5-point ordinal rating scale of “Never,” “Rarely,” “Sometimes,” “Often,” and “Always.” Raw score totals are converted to an item response theory-based T-scores [11]. | X |  | X | X | X |
| Well-being | PROMIS Global Health | The PROMIS Global Health-10 consists of 10 items and assesses generic Health-related Quality of Life [12]. | X |  | X | X | X |
| Social support | PROMIS-57 Profile v2.0 4a | The PROMIS Short Form Emotional support, Informational support and Instrumental support are part of the PROMIS-57 health profile v2.0 Each scale consists of 4 items and assesses perceived support. For each scale, respondents are asked if they have experienced support, using a 5-point ordinal rating scale of “Never,” “Rarely,” “Sometimes,” “Often,” and “Always.” Raw score totals are converted to an item response theory-based T-scores [11]. | X |  | X | X | X |
| Vigilance and interpretation of physical sensations | Bodily Threat Monitoring Scale | Vigilance and interpretation of physical sensations will be assessed with the Bodily Threat Monitoring Scale (BTMS). The BTMS comprises 19 questions that assess the extent to which individuals monitor and interpret bodily sensations as signals of bodily threat (e.g. “I keep track of my bodily sensations to make sure I won’t miss if something is wrong”) [13]. | X |  | X | X | X |
| Depression | PROMIS-57 Profile v2.01 | The PROMIS Short Form v2.01 depression and anxiety scales are part of the PROMIS-57 health profile v2.01. Both consist of 8 items and assess respectively low and depressed mood, or anxiety. For each scale, respondents are asked how often in the past 7 days they have experienced specific depression/anxiety symptoms, using a 5-point ordinal rating scale of “Never,” “Rarely,” “Sometimes,” “Often,” and “Always.” Raw score totals are converted to an item response theory-based T-score [11]. | X |  | X | X | X |
| Anxiety |  |  | X |  | X | X | X |
| Determinants for PA   (beliefs about consequences and capabilities regarding PA) | Determinants of PA Questionnaire | Determinants for PA will be measured via the **Determinants of PA Questionnaire** (DPAQ), assessing several determinants, amongst which self-efficacy, risk perceptions, outcome expectancies, intention, action planning, coping planning and self-monitoring) about PA [14]. Each construct is assessed with a minimum of 3 items. The content validity of this instruments has been investigated using individuals with lived experience (i.e. chronic pain) and domain experts using the discriminant content validity method and using cognitive interviews [15]. Participants are instructed to respond to the items by using a 5-point response scale (1: totally not agree, 5: totally agree). | X |  | X | X | X |
| **Health-economic outcomes** | | | | | | | |
| Return to work rate | Self-composed | A self-composed questionnaire will be used to determine whether participants are working, working part-time/full-time, or working in an adapted work environment. |  |  | X | X | X |
| Medical costs of medical consumption | Medical Consumption Questionnaire (MCQ) | The Medical Consumption Questionnaire [16] is a generic instrument for measuring direct medical costs of a patients’ total medical consumption, including additional diagnostics, consultations surgery including stay in hospitals, physiotherapy, medication and aids prescribed by the general practitioner as well as medication and aids purchased by the patients themselves. |  |  | X | X | X |
| Indirect costs related to disease outside health care | Productivity Cost Questionnaire | The Productivity Cost Questionnaire [16] is a generic instrument to obtain data regarding the indirect costs outside health care, but related to the disease (e.g. the costs due to absence of work and possible decreased productivity at a paid job or at an unpaid job). |  |  | X | X | X |
| Incremental cost-effectiveness ratio  Quality-Adjusted Life-Years | EQ-5D-5L | The EQ-5D-5L questionnaire [17] will be used in combination with the Belgian value set to obtain the health-related quality of life. This enables the use of Quality-Adjusted Life-Years (QALY) as outcome of each intervention arm. |  |  | X | X | X |
| **Trial-related outcomes** | | | | | | | |
| Beliefs about the efficacy of treatment | Patient Global Impression of Change | The Patient Global Impression of Change (PGIC) measure has frequently been used as an indicator of meaningful change in treatments for chronic pain [18]. Patients rate 4 items assessing their impression of specific changes in: pain, PA, daily functioning, and fatigue. |  |  | X |  |  |
| Compliance – adherence to eHealth self-management intervention | number of logins, sessions completed, features accessed, and time spent actively using the program |  |  |  | X* |  |  |
| Compliance – adherence to face-to-face self-management intervention | number of educational sessions,  number of PA coaching sessions |  |  |  | X** |  |  |
| Reason for discontinuation |  |  |  |  | X | X | X |
| Adverse event assessment |  |  |  |  | X | X | X |

* *^1^Informed Consent be obtained prior to performing any other trial-related procedures*

*** *Only participants in the eHealth self-management group will need to complete this questionnaire.*

*** Only participants in the face-to-face self-management group will need to complete this questionnaire.*

The self-report instruments of the PROMIS system are widely used across the world and are well-validated in individuals with medical conditions ([www.healthmeasure.net/](http://www.healthmeasure.net/)).

**References**

1. Pelikan, J.M., et al., *8: Measuring health literacy in Europe: Introducing the European Health Literacy Survey Questionnaire (HLS-EU-Q)*

*International Handbook of Health Literacy*. 2019, Policy Press: Bristol, UK. p. 115-138.

2. van der Vaart, R., et al., *Does the eHealth Literacy Scale (eHEALS) measure what it intends to measure? Validation of a Dutch version of the eHEALS in two adult populations.* J Med Internet Res, 2011. **13**(4): p. e86.

3. Van der Gucht, E., et al., *The Dutch language version of the Pain Disability Index (PDI-DLV): psychometric properties in breast cancer patients.* Physiother Theory Pract, 2022: p. 1-15.

4. Jensen, M.P., J.A. Turner, and J.M. Romano, *Pain belief assessment: A comparison of the short and long versions of the surgery of pain attitudes.* The Journal of Pain, 2000. **1**(2): p. 138-150.

5. Osman, A., et al., *The Pain Catastrophizing Scale: Further Psychometric Evaluation with Adult Samples.* Journal of Behavioral Medicine, 2000. **23**(4): p. 351-365.

6. Meeus, M., *Development and properties of the Dutch Neurophysiology of Pain Test in patients with chronic fatigue syndrome.* J Musciloskelet Pain, 2010. **18**(1): p. 58-65.

7. Margolis, R.B., R.C. Tait, and S.J. Krause, *A rating system for use with patient pain drawings.* Pain, 1986. **24**(1): p. 57-65.

8. Bouhassira, D., et al., *Development and validation of the Neuropathic Pain Symptom Inventory.* Pain, 2004. **108**(3): p. 248-57.

9. Pfister, T., et al., *Comparison of two accelerometers for measuring physical activity and sedentary behaviour.* BMJ Open Sport Exerc Med, 2017. **3**(1): p. e000227.

10. Cane, D. and D. Mazmanian, *Development and Initial Evaluation of the Patterns of Activity Measure-Pain Short Form.* Clin J Pain, 2020. **36**(9): p. 675-682.

11. Elsman, E.B.M., et al., *Measurement properties of the Dutch PROMIS-29 v2.1 profile in people with and without chronic conditions.* Qual Life Res, 2022. **31**(12): p. 3447-3458.

12. Pellicciari, L., et al., *Psychometric properties of the patient-reported outcomes measurement information system scale v1.2: global health (PROMIS-GH) in a Dutch general population.* Health and Quality of Life Outcomes, 2021. **19**(1): p. 226.

13. Heathcote, L.C., et al., *The bodily threat monitoring scale: Development and preliminary validation in adult and childhood cancer survivors.* Psychooncology, 2023. **32**(12): p. 1885-1894.

14. Taylor, N., R. Lawton, and M. Conner, *Development and initial validation of the determinants of physical activity questionnaire.* International Journal of Behavioral Nutrition and Physical Activity, 2013. **10**(1): p. 74.

15. Poppe, L., et al., *Efficacy of a Self-Regulation-Based Electronic and Mobile Health Intervention Targeting an Active Lifestyle in Adults Having Type 2 Diabetes and in Adults Aged 50 Years or Older: Two Randomized Controlled Trials.* J Med Internet Res, 2019. **21**(8): p. e13363.

16. Bouwmans, C.A.M., et al. *Handleiding iMTA Medical Cost Questionnaire (iMCQ)*. 2013.

17. Bouckaert, N., et al., *An EQ-5D-5L Value Set for Belgium.* PharmacoEconomics - Open, 2022. **6**(6): p. 823-836.

18. Scott, W. and L.M. McCracken, *Patients' impression of change following treatment for chronic pain: global, specific, a single dimension, or many?* J Pain, 2015. **16**(6): p. 518-26.
