# Supplementary material for: Study protocol for a pragmatic randomised controlled trial in Belgian primary care and hospital settings on the effectiveness of an eHealth self-management support programme consisting of pain education and coaching of activity needs in breast cancer survivors with persistent pain: the PECAN trial
Source: BMJ Open. 2025 Aug 22;15(8):e099241. doi: 10.1136/bmjopen-2025-099241 (PMC12374636; doi:10.1136/bmjopen-2025-099241)
Supplement: online supplemental file 4 [file bmjopen-15-8-s004.docx]

Effectiveness of an eHealth self-management support program for persistent pain after breast cancer treatment

Process evaluation

The general aim of the PEACAN project is to investigate the effectiveness of an eHealth self-management support program for pain-related disability in breast cancer survivors with persistent pain. The primary scientific objective of PEACAN is to determine the effectiveness of an eHealth self-management support program for persistent pain after breast cancer treatment compared to:

1) usual care (i.e. superiority of the eHealth self-management support program) and

2) a comprehensive pain rehabilitation program delivered face-to-face in a physical therapy setting.

The objective of the PEACAN process evaluation is to assess a) implementation fidelity of the intervention across the three arms; b) to assess contextual factors affecting implementation and outcomes, and c) to assess influencing factors, assessing scalability of the intervention meaning the feasibility of integration in routine health care processes.

**Interventions**

PEACAN has three arms each with their own intervention-components, listed in table below.

|  | **eHealth self-management** | **Face-to-face self-management** | **Brochure self-management** |
| --- | --- | --- | --- |
| **Core elements of intervention** | -Participants receive access code  -Education sessions: 25  - Physical activity coaching sessions: 4 phases | -Referral primary care physical therapist  -Education sessions: 3  - Physical activity coaching sessions: 6 | - Brochure is sent out to the participant  - Recommendation to consult health care provider |

**Methods:** Goal a) and b) will be assessed during the implementation phase. Goal c) will be assessed after the implementation and outcome evaluation have been finalized.

**a) Implementation fidelity**

Implementation fidelity assesses whether the intervention was delivered to the participant as intended looking at the following elements:

- Delivery: the intervention components that have been delivered by the provider/program
- Dosage received: the dosage of intervention components the participants have received
- Reach: number of participants that received the full intervention

|  | **eHealth self-management** | **Face-to-face self-management** | **Brochure self-management** |
| --- | --- | --- | --- |
| **Delivery measure** | -Nr of eHealth-programme access codes handed out  - Nr of educational sessions delivered to each patient  - Nr of physical activity coaching sessions delivered to each patient | - Nr of referrals written to primary care physical therapist  - Nr of educational sessions delivered to each patient  - Nr of physical activity coaching sessions delivered to each patient | - Nr of brochures sent out |
| **Dosage received** | - Nr of patients that opened the eHealth programme at least once  - Nr of times that each patient opened the program  - Time spent on educational sessions by each patient  - Number of features accessed  - Time spent on physical activity coaching sessions by each patient | - Nr of patients that had at least one consultation with primary care physical therapist  - Nr of patients that has at least the educational sessions at primary care physical therapist | - Nr of patients that remembered to have received the brochure  - Nr of people that have consulted a health care provider for pain |

Data collection from start of intervention.

**b) Contextual factors** that affect implementation and outcomes

- healthcare providers and patients (nano-level)
  - referring health care provider: general practitioner, oncologist, other
  - patient: vulnerable situation
- individual health care organisation (micro-level)
  - GP practice:
    - fee-for-service/NewDeal/forfetary system
  - primary care physical therapist under same roof or different location
- collaborative structures for transmural care first-second line (meso-level)
  - existing collaborations between referring provider and primary care physical therapist

Dat collection through survey interviews with healthcare providers and patients (from the start of intervention).

**c) Scalability of the intervention**

Meaning the feasibility of integration in routine health care processes.

For this, observations and interviews will be organised with the key informants and stakeholders at health care organisation level and umbrella organisations and policy makers. For instance Domus Medica, hospital management, RIZIV, Agentschap Zorg en Gezondheid, Ministry of Health.

These observations and interviews will be organised during (from month 25) and shortly after the intervention (month 33-36).
