# Supplementary material for: Study protocol for a pragmatic randomised controlled trial in Belgian primary care and hospital settings on the effectiveness of an eHealth self-management support programme consisting of pain education and coaching of activity needs in breast cancer survivors with persistent pain: the PECAN trial
Source: BMJ Open. 2025 Aug 22;15(8):e099241. doi: 10.1136/bmjopen-2025-099241 (PMC12374636; doi:10.1136/bmjopen-2025-099241)
Supplement: Supplementary file 5 [file bmjopen-2025-099241.reviewer_comments.pdf]

PEER REVIEW HISTORY

BMJ Open publishes all reviews undertaken for accepted manuscripts. Reviewers are asked to complete a checklist review form and are provided with free text boxes to elaborate on their assessment. These free text comments are reproduced below.

ARTICLE DETAILS

Title (Provisional)

Study protocol for a pragmatic randomized controlled trial in Belgian primary care and hospital settings on the effectiveness of an eHealth self-management support program consisting of Pain Education and Coaching of Activity Needs in breast cancer survivors with persistent pain: the PECAN trial

Authors

De Groef, An; Dams, Lore; Moseley, G. Lorimer; Heathcote, Lauren; Wiles, Louise K; Catley, Mark; Vogelzang, Anna; Hibbert, Peter; Morlion, Bart; Van Overbeke, Marthe; Tack, Emma; Van Dijck, Sophie; Devoogdt, Nele; Gursen, Ceren; De Paepe, Annick L; Mertens, Michel; van Olmen, Josefien; Willem, Lander; Tjalma, W; Nevelsteen, Ines; Neven, P.; Vanhoudt, Rani; Wildemeersch, Davina; De Backere, Femke; Fieuws, Steffen; Crombez, Geert; Meeus, Mira

---

VERSION 1 - REVIEW

**Reviewer**	**1**
**Name**	**Fernández-Lao, Carolina**
**Affiliation**	**University of Granada, Physiotherapy Department**
**Date**	**12-Feb-2025**
**COI**	**None**

I have to thank for the opportunity of review this interesting and well written research protocol. It approaches to an important topic and try to address to the needs previously detected on the aimed population. I find it very positive that this research group is developing a new eHealth tool based on the limitations or issues identified in previous important studies.

The design seems adequate and well specified.

It has many positive aspects as the use of validated scales (PDI, SOPA-B, PCS, PROMIS) and the assessment at multiple time points.

--However too many measures may increase the burden on participants. It would be interesting to prioritize the most relevant ones and justify whether some could be removed, just in case of the mentioned burden.

--A non-inferiority margin of 5 points is set on the Pain Disability Index (PDI). It should be better justified/referenced whether this margin represents a clinically significant change and not just a statistical one.

--It would be helpful to comment on the clinical relevance and significance at the end of the manuscript, taking into account the wide previous experience in the field of research of this research group.

**Reviewer**	**2**
**Name**	**Andrews, Nicole Emma**
**Affiliation**	**The University of Queensland, RECOVER Injury Research Centre**
**Date**	**12-Apr-2025**
**COI**	**None**

This is an interesting study and I wish the authors the best with conducting this trial.

I have a few minor suggestions to improve the study protocol.

One of the main concerns with a trial like this is that participants won't engage well with the eHealth intervention. I would be good to have some added information in the introduction concerning acceptability and engagement with chatbots and eHealth interventions and comment on any existing or similar chatbots and eHealth interventions in pain.

With the analysis, I was curious why the non-inferiority analysis would not be conducted if superiority was not established. Is this standard practice? Wouldn't it still be valuable for the scientific community to know the results of the non-inferiority analysis regardless? Could you add to your rationale here? Please also add the rationale of the non-inferiority analysis being based on an as treated analysis set.

More details are needed on the health economic analysis. Please add a section about this to the main text.

VERSION 1 - AUTHOR RESPONSE

Reviewer: 1
Dr. Carolina Fernández-Lao, University of Granada Comments to the Author:
I have to thank for the opportunity of review this interesting and well written research protocol. It approaches to an important topic and try to address to the needs previously detected on the aimed population. I find it very positive that this research group is developing a new eHealth tool based on the limitations or issues identified in previous important studies. The design seems adequate and well specified. It has many positive

aspects as the use of validated scales (PDI, SOPA-B, PCS, PROMIS) and the assessment at multiple time points.
RE: Thank you for your positive and encouraging words!

--However too many measures may increase the burden on participants. It would be interesting to prioritize the most relevant ones and justify whether some could be removed, just in case of the mentioned burden.
RE: Thank you for raising this important concern. We are aware of the large number of measurements and the potential burden on participants. The selection of outcomes was guided by a program theory outlining the possible mechanisms of action of the eHealth intervention (see Section 'Outcomes'). This process led to the current set of outcome measures. During trial development, patients were consulted to assess the relevance and feasibility of these outcomes (see Section 'Patient and Public Involvement in Trial Design'). Based on their feedback, the selected outcomes were considered both relevant and manageable. Therefore, we are confident that the burden on participants is reasonable.

--A non-inferiority margin of 5 points is set on the Pain Disability Index (PDI). It should be better justified/referenced whether this margin represents a clinically significant change and not just a statistical one.
RE: Thank you for this important point. The non-inferiority margin of 5 points on the Pain Disability Index (PDI) was deliberately chosen to be conservative and well below the threshold of what is considered a minimal clinically important change (MCIC). Existing literature suggests that the MCIC for the PDI ranges between 8.5 and 13 points in various patient populations with chronic pain (Ostelo et al., 2011; De Vet et al., 2018). By selecting a 5-point margin—significantly lower than these established thresholds—we ensure that any potential di\erence between the eHealth and face-to-face interventions remains clinically negligible from the patient's perspective. This margin therefore reflects not only statistical considerations but also safeguards clinical relevance.

--It would be helpful to comment on the clinical relevance and significance at the end of the manuscript, taking into account the wide previous experience in the field of research of this research group.
*RE: Thank you for pointing out a conclusion at the end of the manuscript was missing. We have added a conclusion section summarizing the trial objectives and relevance: "*This protocol describes a pragmatic, three-arm randomized controlled trial evaluating the egectiveness of an innovative eHealth self-management program for persistent pain after breast cancer treatment. By comparing the eHealth intervention to both usual care and a face-to-face program, the study aims to assess its superiority and non-inferiority, respectively. The intervention is grounded in a biopsychosocial model and leverages pain science education and behavior change principles to promote an active lifestyle. Delivered via a chatbot, the program is designed to be accessible, scalable, and cost-egective. Its digital format ogers the potential to overcome common barriers to pain rehabilitation, such

as limited availability of specialized care and time constraints. The trial is conducted in both hospital and primary care settings to enhance external validity. Pain-related disability at six months is the primary outcome, supported by a broad set of secondary outcomes assessing physical, psychological, and health-economic egects. Patient input informed the design, ensuring relevance and feasibility. Results from this trial will contribute valuable evidence on the role of digital self-management in cancer-related pain rehabilitation and inform future implementation strategies."

Reviewer: 2
Dr. Nicole Emma Andrews, The University of Queensland, Metro North Hospital and Health Service
Comments to the Author:
This is an interesting study and I wish the authors the best with conducting this trial. *RE: Thank you for your kind words and encouragements.*

I have a few minor suggestions to improve the study protocol. One of the main concerns with a trial like this is that participants won't engage well with the eHealth intervention. I would be good to have some added information in the introduction concerning acceptability and engagement with chatbots and eHealth interventions and comment on any existing or similar chatbots and eHealth interventions in pain.
RE: We acknowledge this important aspect of an eHealth intervention using a chatbot may not have been highlighted enough in the introduction. We have added the following sentences showing evidence for good engagement in eHealth interventions using a chatbot:"
Studies testing chatbot-based eHealth interventions in breast cancer populations and chronic pain settings report high patient engagement, satisfaction, and usability, suggesting that chatbots can serve as egective, scalable tools for delivering personalized support outside traditional care settings (Chaix et al 2019, Tawfik et al 2023, Sandra Hauser-Ulrich et al 2020)

With the analysis, I was curious why the non-inferiority analysis would not be conducted if superiority was not established. Is this standard practice? Wouldn't it still be valuable for the scientific community to know the results of the non-inferiority analysis regardless? Could you add to your rationale here? Please also add the rationale of the noninferiority analysis being based on an as treated analysis set.
RE: Thank you for this thoughtful question. We agree that non-inferiority findings can provide valuable insight, even if superiority is not established. However, our trial is designed with a hierarchical testing structure, where the superiority comparison (eHealth vs. usual care) is tested first. This approach was chosen to preserve the family-wise error rate and to maintain a clear interpretation of results, following guidance on multiple hypothesis testing in clinical trials (Wiens BL, 2002). Under this structure, the non-inferiority analysis (eHealth vs. face-to-face) is only conducted if superiority is demonstrated to avoid inflating the type I error rate.

That said, we acknowledge the scientific interest in the non-inferiority comparison regardless of superiority outcomes. To address this, we will transparently report the results of the non-inferiority comparison as a secondary exploratory analysis if superiority is not achieved. However, any such findings will be interpreted cautiously and not used to support formal claims of non-inferiority unless the superiority threshold is met.

The non-inferiority comparison will indeed be based on an as-treated analysis set. This choice reflects established guidance for non-inferiority trials, where an as-treated or perprotocol analysis is preferred to avoid dilution of group di\erences that may occur due to non-adherence or protocol deviations (Piaggio et al., 2006). This analytical approach provides a more conservative and accurate estimate of the treatment e\ect for noninferiority purposes, ensuring that any conclusion of equivalence is robust and not driven by cross-over or dropouts.

More details are needed on the health economic analysis. Please add a section about this to the main text.
RE: Thank you for this suggestion. We acknowledge that the health-economic analysis should be explained in more detail in the text. Therefore the following was added: "For the health-economic analysis, a self-composed questionnaire will be used to determine whether participants are working, working part-time/full-time, or working in an adapted work environment.
Socio-economic outcomes will be evaluated using: (1) the **Medical Consumption Questionnaire** (63), a generic instrument for measuring direct medical costs of patients' total medical consumption, including additional diagnostics, consultations surgery including stay in hospitals, physiotherapy, medication and aids prescribed by the general practitioner as well as medication and aids purchased by the patients themselves; and (2) the **Productivity Cost Questionnaire** (64) to obtain data regarding the indirect costs outside health care, but related to the disease (e.g. the costs due to absence of work and possible decreased productivity at a paid job or at an unpaid job). These two questionnaires are understandable, easy-to-use and generate valid data (65, 66). The combination of these questionnaires is advised by the Institute for Medical Technology Assessment, Erasmus University Rotterdam (the Netherlands) (67), and has served in a large number of high-quality cost-egectiveness trials. In addition, a generic health-related quality of life questionnaire, the **EQ-5D-5L questionnaire** will be administered. With this information the incremental cost-egectiveness ratio (ICER) will be calculated as general outcome of economic benefit (cost-egectiveness) and Quality-Adjusted Life-Years (QALY) as outcome of enhanced quality of life (cost-utility analysis)."
